# Synthesis and Fluorescent Properties of Multi-Functionalized C_70_ Derivatives of C_70_(OCH_3_)_10_[C(COOEt)_2_] and C_70_(OCH_3_)_10_[C(COOEt)_2_]_2_

**DOI:** 10.3390/nano12091426

**Published:** 2022-04-22

**Authors:** Ke Luan, Lu Wang, Fang-Fang Xie, Bin-Wen Chen, Zuo-Chang Chen, Lin-Long Deng, Su-Yuan Xie, Lan-Sun Zheng

**Affiliations:** 1 State Key Laboratory for Physical Chemistry of Solid Surfaces, Collaborative Innovation Center of Chemistry for Energy Materials, Department of Chemistry, College of Chemistry and Chemical Engineering, Xiamen University, Xiamen 361005, China; luanke9411@163.com (K.L.); 20520201151919@stu.xmu.edu.cn (L.W.); fangfangxie0707@163.com (F.-F.X.); cbw15959235525@163.com (B.-W.C.); zcchem@126.com (Z.-C.C.); syxie@xmu.edu.cn (S.-Y.X.); lszheng@xmu.edu.cn (L.-S.Z.); 2 Pen-Tung Sah Institute of Micro-Nano Science and Technology, Xiamen University, Xiamen 361005, China

**Keywords:** fullerene, multi-functionalization, fluorescence

## Abstract

Due to the partially reduced π-conjugation of the fullerene cage, multi-functionalized fullerene derivatives exhibit remarkable fluorescent properties compared to pristine fullerenes, which have high potential for application in organic light-emitting diodes (OLEDs). In this study two multi-functionalized C_70_ derivatives, C_70_(OCH_3_)_10_[C(COOEt)_2_] and C_70_(OCH_3_)_10_[C(COOEt)_2_]_2_, with excellent fluorescence properties, were designed and synthesized. Compared with C_70_(OCH_3_)_10_ containing a single kind of functional group, both the C_70_(OCH_3_)_10_[C(COOEt)_2_] and C_70_(OCH_3_)_10_[C(COOEt)_2_]_2_ exhibited enhanced fluorescence properties with blue fluorescence emission. The fluorescence quantum yields of the C_70_(OCH_3_)_10_[C(COOEt)_2_] and C_70_(OCH_3_)_10_[C(COOEt)_2_]_2_ were 1.94% and 2.30%, respectively, which were about ten times higher than that of C_70_(OCH_3_)_10_. The theoretical calculations revealed that the multi-functionalization of the C_70_ increased the S_1_–T_1_ energy gap, reducing the intersystem crossing efficiency, resulting in the higher fluorescence quantum yield of the C_70_ derivatives. The results indicate that multi-functionalization is a viable strategy to improve the fluorescence of fullerene derivatives.

## 1. Introduction

Fluorescence studies on fullerenes and their derivatives have attracted great interest from researchers [1,2,3,4,5,6,7,8,9,10,11,12,13], who can not only offer vital information on the excited electronic structures of fullerenes and their derivatives, but can also assess their potential applications in organic electronic devices [14,15]. Due to the renowned electron-accepting ability and small reorganization energy of symmetric fullerenes [16,17], the transition from S_0_ to S_1_ is forbidden, and the intersystem crossing (ISC) efficiency from S_1_ to T_1_ is very high (close to 100%) [18]. Pristine fullerenes exhibit poor fluorescence properties, such as low-fluorescence quantum yields (*Φ* of ca. 0.03% for C_60_ and ca. 0.06% for C_70_ in toluene) and short fluorescence lifetimes (τ of 1.2 ns for C_60_ and 0.67 ns for C_70_) [19,20,21,22]. The functionalization of fullerene is a valid way to increase electronic transition forbiddance and the S_1_–T_1_ energy gap by lowering the symmetry of the fullerene. However, the fluorescence of mono-, bis-, and tris-adducts of fullerene derivatives is still extremely weak, since these adducts cannot effectively destroy the symmetric structure of fullerenes [23]. Multi-functionalization with higher adducts has been proven to be an effective methodology to fine-tune the fluorescence properties of fullerene derivatives. For instance, Rubin et al. reported a hexa-adduct of C_60_ that exhibited much-improved fluorescence intensity [24]. Multi-functionalized C_60_ derivatives with excellent fluorescence properties were prepared by Nakamura et al. [25,26,27]. Compared with the studies on the fluorescence properties of C_60_ derivatives, there are few studies on the fluorescence properties of C_70_ derivatives [28].

Herein, we report the synthesis and fluorescence properties of two multi-functionalized C_70_ derivatives, C_70_(OCH_3_)_10_[C(COOEt)_2_] and C_70_(OCH_3_)_10_[C(COOEt)_2_]_2_. By carefully controlling the molar ratio of the reactants, C_70_(OCH_3_)_10_[C(COOEt)_2_] and C_70_(OCH_3_)_10_[C(COOEt)_2_]_2_ can be readily synthesized from C_70_(OCH_3_)_10_ by using the Bingel–Hirsch reaction with high selectivity. Due to the reduced π-conjugated system of C_70_, the fluorescence quantum yield of C_70_(OCH_3_)_10_[C(COOEt)_2_] and C_70_(OCH_3_)_10_[C(COOEt)_2_]_2_ was about ten times higher than that of C_70_(OCH_3_)_10_. The results provide a method for synthesizing fullerene derivatives with excellent fluorescence, offering valuable materials for organic light-emitting diodes.

## 2. Materials and Methods

### 2.1. Materials and Synthesis

C_70_, iodine monochloride (ICl), silver perchlorate, diethyl bromomalonate, and 1,8-diazabicyclo[5.4.0]undec-7-ene (DBU) were purchased from commercial suppliers and used as received without further purification. Solvents were distilled and dried by standard procedures.

C_70_Cl_10_ and C_70_(OCH_3_)_10_ were prepared according to the procedure in the literature [28,29].

C_70_(OCH_3_)_10_[C(COOEt)_2_]: Diethyl bromomalonate (12 mg, 0.05 mmol) and DBU (16 mg, 0.1 mmol) were added to a solution of C_70_(OCH_3_)_10_ (58 mg, 0.05 mmol) in anhydrous toluene (50 mL). The mixture was stirred overnight under atmosphere at room temperature. The solvent was removed under reduced pressure and the crude product was purified by column chromatography over silica gel with toluene/acetate (10:1) as the eluents to produce C_70_(OCH_3_)_10_[C(COOEt)_2_] as a pale-yellow solid (22 mg, 33%). ^1^H NMR (500 MHz, CDCl_3_, ppm): *δ* 4.21 (q, *J* = 7.0 Hz, 4H), 3.98 (s, 6H), 3.93 (s, 12H), 3.86 (s, 6H), 3.75 (s, 6H), and 1.22 (t, *J* = 7.0 Hz, 6H). ^13^C NMR (500 MHz, CDCl_3_, ppm): *δ* 163.56, 153.40, 153.07, 151.78, 151.29, 150.78, 150.73, 149.91, 149.40, 148.90, 148.54, 148.42, 148.24, 148.22, 148.11, 147.88, 147.79, 146.61, 146.49, 146.13, 145.41, 145.12, 143.71, 142.96, 139.00, 138.83, 138.58, 137.46, 135.93, 134.20, 129.04, 128.23, 86.18, 81.21, 81.03, 80.81, 80.69, 67.79, 65.89, 63.10, 56.18, 56.11, 55.94, 55.91, 55.85, 43.47, and 14.03. ESI-FT-ICR-HRMS C_87_H_40_O_14_ [M+Na]^+^ *m/z* calculated 1331.2310 found 1331.2311.

The C_70_(OCH_3_)_10_[C(COOEt)_2_]_2_: Diethyl bromomalonate (48 mg, 0.2 mmol) and DBU (60 mg, 0.4 mmol) were added to a solution of C_70_(OCH_3_)_10_ (58 mg, 0.05 mmol) in anhydrous toluene (50 mL). The mixture was stirred overnight under atmosphere at room temperature. The solvent was removed under reduced pressure and the crude product was purified by column chromatography over silica gel with toluene/acetate (5:1) as the eluents to afford C_70_(OCH_3_)_10_[C(COOEt)_2_]_2_ as a light-yellow solid (29 mg, 39%). ^1^H NMR (500 MHz, CDCl_3_, ppm): *δ* 4.33 (m, 8H), 4.00–3.77 (m, 30H), and 1.38–1.30 (m, 12H). ^13^C NMR (500 MHz, CDCl_3_, ppm): *δ* 163.70, 163.67, 163.64, 163.21, 154.21, 153.22, 151.89, 151.80, 151.39, 151.34, 150.58, 150.33, 150.00, 149.62, 149.02, 148.90, 148.59, 148.31, 147.83, 147.53, 147.24, 146.91, 146.62, 146.52, 146.44, 146.30, 145.85, 145.60, 145.30, 145.26, 145.01, 144.60, 144.47, 143.67, 142.71, 139.95, 139.66, 139.12, 138.89, 138.14, 137.77, 137.52, 136.59, 136.24, 135.58, 134.38, 133.50, 130.01, 85.49, 84.51, 81.06, 80.94, 80.86, 80.80, 80.76, 80.70, 67.89, 67.80, 63.10, 63.07, 62.94, 62.88, 55.97, 55.93, 55.87, 55.79, 55.71, 55.68, 55.20, 43.44, 41.00, and 14.06. ESI-FT-ICR-HRMS C_94_H_50_O_18_ [M+Na]^+^ *m/z* calculated 1490.3847 found 1490.2916.

### 2.2. Characterization

^1^H NMR, ^13^C NMR, and 2D NMR spectra were recorded using Bruker AVⅢ500 spectrometers (Bruker, Billerica, MA, USA). High-resolution mass spectra (HRMS) were recorded on Agilent G6545XT mass spectrometers (Agilent, Santa Clara, CA, USA). UV-vis absorption spectra in solution were recorded using an Agilent Cary 5000 spectrophotometer (Agilent, Santa Clara, CA, USA). The spectra were measured in quartz glass cuvettes using spectroscopic grade solvents. Fluorescence spectroscopy in solution was carried out with an FLS980 spectrometer (Edinburgh Instruments, Livingston, UK). Time-resolved measurements were performed with a PS laser diode and a TCSPC detection unit. Single-crystal X-ray data were collected on a Rigaku Xtalab Synergy diffractometer (Rigaku, Tokyo, Japan). Using Olex2 [30], the initial structure was solved with the SHELX-XT structure solution program using direct method and refined with the XL refinement package using least-squares minimization.

## 3. Results and Discussion

**Scheme 1:**
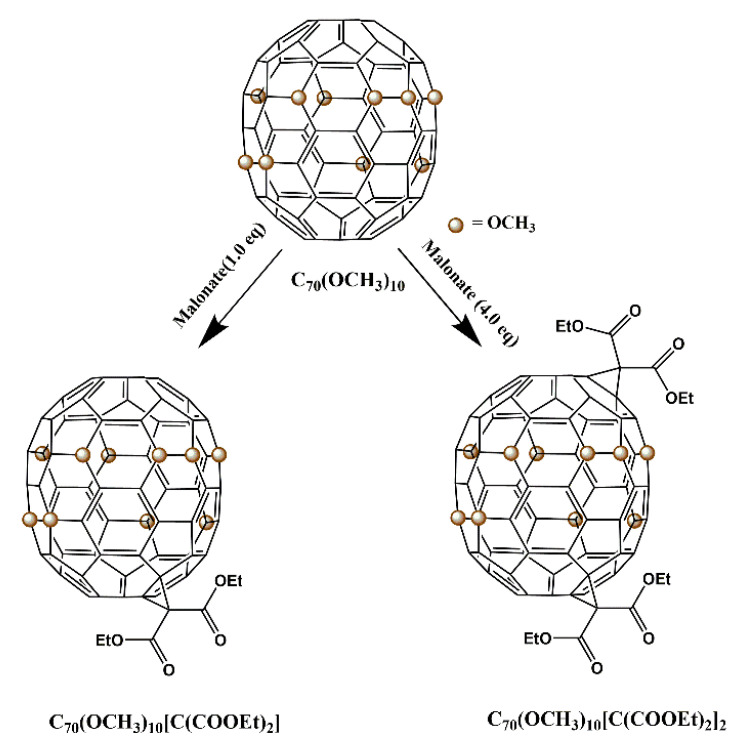
Synthesis of C_70_(OCH_3_)_10_[C(COOEt)_2_] and C_70_(OCH_3_)_10_[C(COOEt)_2_]_2_.

As shown in Scheme 1, the C_70_(OCH_3_)_10_[C(COOEt)_2_] and C_70_(OCH_3_)_10_[C(COOEt)_2_]_2_ were synthesized from C_70_(OCH_3_)_10_ by Bingel–Hirsch reaction. The deca-adduct C_70_ derivative C_70_(OCH_3_)_10_ was readily prepared by treating the C_70_Cl_10_ with anhydrous methanol in the presence of silver perchlorate. C_70_(OCH_3_)_10_[C(COOEt)_2_] and C_70_(OCH_3_)_10_[C(COOEt)_2_]_2_ can be readily synthesized with high selectivity by carefully controlling the molar ratio of the reactants. Their molecular structures were confirmed by ^1^H, ^13^C NMR spectroscopy and high-resolution mass spectrometry (Figures S1–S10). The two-dimensional correlated spectroscopy (COSY) showed that there was mutual coupling of the protons between the methyl and the methylene in the ethyl malonate of both the C_70_(OCH_3_)_10_[C(COOEt)_2_] and the C_70_(OCH_3_)_10_[C(COOEt)_2_]_2_ molecules (Figures S4 and S9).

**Figure 1:**
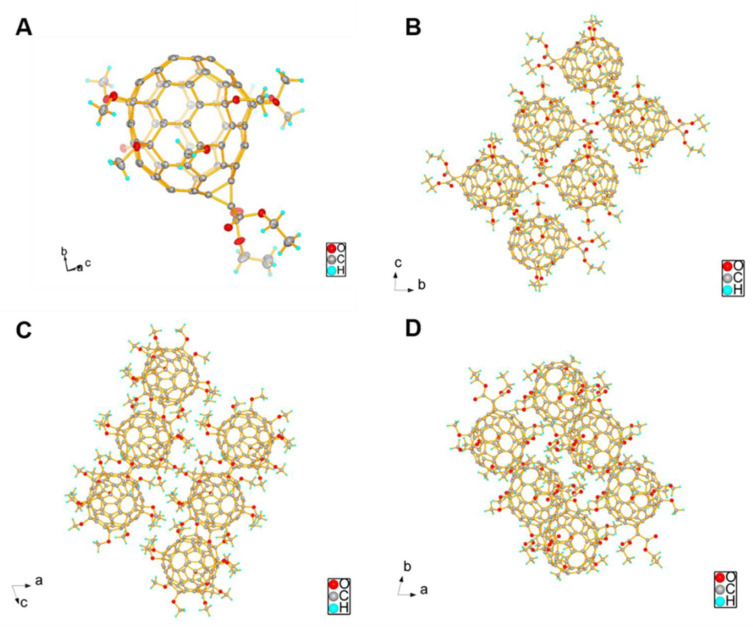
Crystal structure of C_70_(OCH_3_)_10_[C(COOEt)_2_]. (**A**) ORTEP drawing with 50% ellipsoid probability. The molecular packing along a-axis (**B**), b-axis (**C**), and c-axis (**D**).

The structure of the C_70_(OCH_3_)_10_[C(COOEt)_2_] was unambiguously determined by X-ray crystallographic analysis (Figure 1 and Table S1). Single crystals were obtained through the slow diffusion of hexane into a toluene solution of C_70_(OCH_3_)_10_[C(COOEt)_2_]. As shown in Figure 1, all the methoxy groups were distributed on the equatorial region of the C_70_ cage. The malonate group was added to the pole of the C_70_ cage, and the ester groups were pointed in different directions to minimize the steric hindrance. In the crystalline state, the C_70_(OCH_3_)_10_[C(COOEt)_2_] molecules displayed ordered packing in all the directions of the a-, b- and c-axes. Although the single crystal of the C_70_(OCH_3_)_10_[C(COOEt)_2_]_2_ was not obtained, the most favorable structure of C_70_(OCH_3_)_10_[C(COOEt)_2_]_2_ was determined through a series of theoretical calculations (Figures S11–S13). As shown in Figure S14, the two malonate groups were distributed at the two poles of the C_70_ cage. Similarly, all the functionalized groups were oriented in different directions to minimize the steric hindrance.

**Figure 2:**
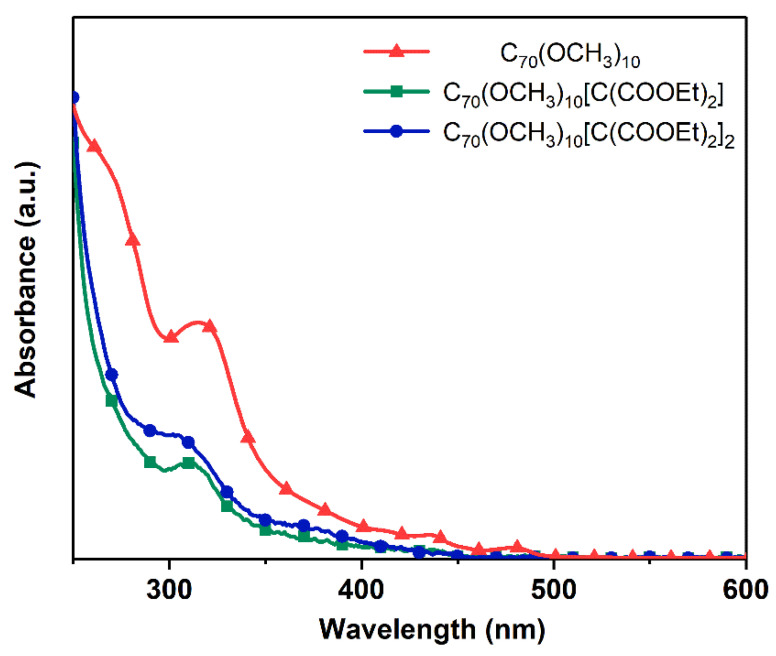
UV-vis absorption spectra of C_70_(OCH_3_)_10_, C_70_(OCH_3_)_10_[C(COOEt)_2_], and C_70_(OCH_3_)_10_[C(COOEt)_2_]_2_ in a 1.0 × 10^−5^ mol/L chloroform solution at room temperature.

The UV-vis absorption spectra of the C_70_(OCH_3_)_10_, C_70_(OCH_3_)_10_[C(COOEt)_2_], and C_70_(OCH_3_)_10_[C(COOEt)_2_]_2_ were measured at room temperature. As shown in Figure 2, the C_70_(OCH_3_)_10_ exhibited two absorption peaks at 435 and 480 nm in the visible region, and one absorption peak at 315 nm in the ultraviolet region. By contrast, there was no absorption peak in the visible region, but there was one absorption peak in the ultraviolet region (313 nm) for C_70_(OCH_3_)_10_[C(COOEt)_2_], which was slightly blue-shifted with respect to the C_70_(OCH_3_)_10_. Similarly, the C_70_(OCH_3_)_10_[C(COOEt)_2_]_2_ showed an absorption peak at 305 nm, which was further blue-shifted compared with that of the C_70_(OCH_3_)_10_[C(COOEt)_2_]. Moreover, a broad shoulder peak around 370 nm was observed for the C_70_(OCH_3_)_10_[C(COOEt)_2_]. The blue-shifting of the absorption peaks of both C_70_(OCH_3_)_10_[C(COOEt)_2_] and C_70_(OCH_3_)_10_[C(COOEt)_2_]_2_ was caused by the decrease in the π-conjugated system of the C_70_ cage, indicating that the energy gap between the S_1_ and S_0_ became lager.

**Figure 3:**
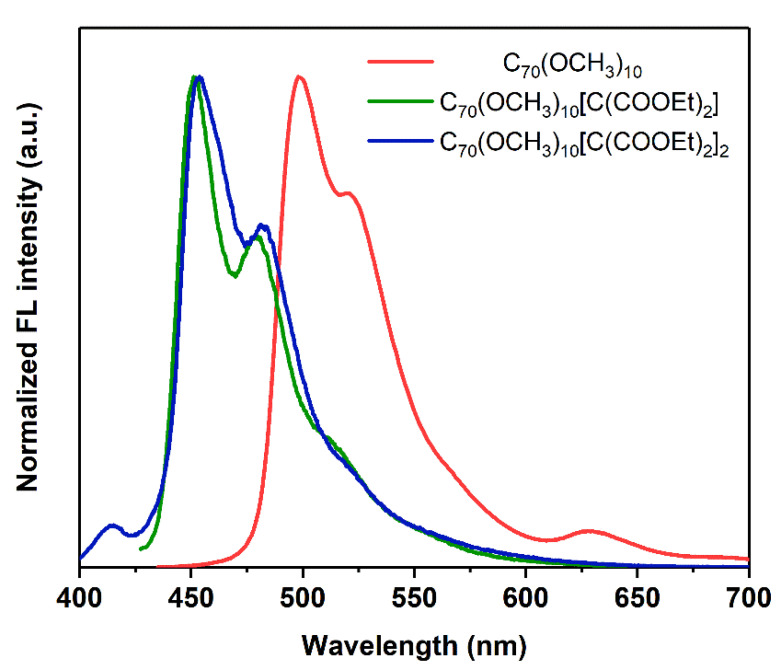
Normalized steady-state fluorescence spectra of C_70_(OCH_3_)_10_, C_70_(OCH_3_)_10_[C(COOEt)_2_], and C_70_(OCH_3_)_10_[C(COOEt)_2_]_2_ at room temperature.

To obtain information about the photophysical properties of the C_70_(OCH_3_)_10_, C_70_(OCH_3_)_10_[C(COOEt)_2_], and C_70_(OCH_3_)_10_[C(COOEt)_2_]_2_, we measured the steady-state fluorescence spectra of these C_70_ derivatives. As shown in Figure 3, the emission peak of the C_70_(OCH_3_)_10_ was 498 nm, with a shoulder peak at 521 nm. The major emission peak at 498 nm was ascribed to the S_1_→S_0_ transition, and the shoulder peak was ascribed to the transition involving the vibronic interactions [4,5]. The fluorescence spectra of the C_70_(OCH_3_)_10_[C(COOEt)_2_] and C_70_(OCH_3_)_10_[C(COOEt)_2_]_2_ were rather similar. The major peaks appeared at 451 and 454 nm for the C_70_(OCH_3_)_10_[C(COOEt)_2_] and the C_70_(OCH_3_)_10_[C(COOEt)_2_]_2_, respectively, while the shoulder peaks were shown at 480, and 481 nm. Obviously, the fluorescence emission peaks of the C_70_(OCH_3_)_10_[C(COOEt)_2_] and C_70_(OCH_3_)_10_[C(COOEt)_2_]_2_ were blue-shifted compared to those of the C_70_(OCH_3_)_10_, indicating that the Bingel–Hirsch reaction can effectively reduce the π-conjugated system of the C_70_ cage [21]. The fluorescence quantum yields of these fullerene derivatives were obtained with integrating spheres. The fluorescence quantum yields of the C_70_(OCH_3_)_10_[C(COOEt)_2_] and C_70_(OCH_3_)_10_[C(COOEt)_2_]_2_ were 1.94, and 2.30%, respectively, which were about ten times higher than that of the C_70_(OCH_3_)_10_ (0.25%). However, the fluorescence quantum yields of both the C_70_(OCH_3_)_10_[C(COOEt)_2_] and the C_70_(OCH_3_)_10_[C(COOEt)_2_]_2_ were not particularly high, which made them difficult to use as fluorescent labels.

**Figure 4:**
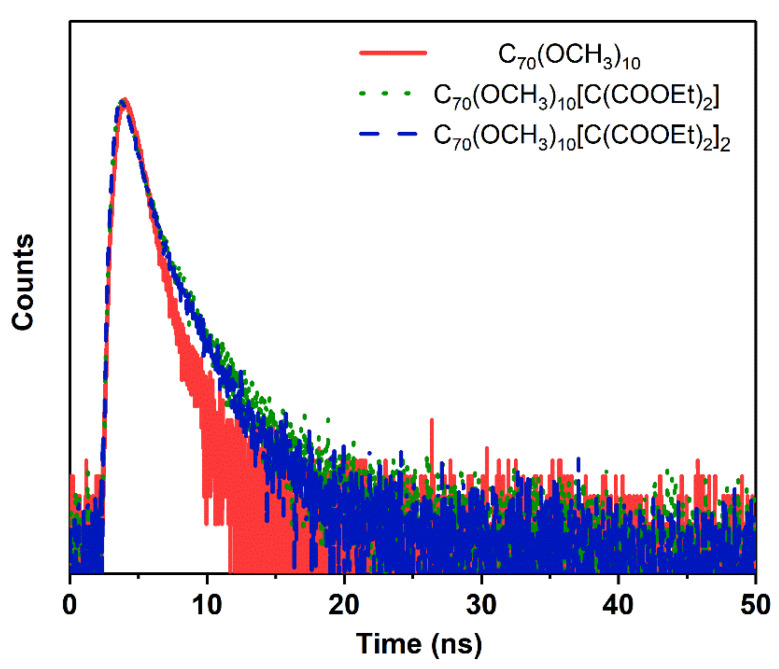
Time-resolved fluorescence decay profiles of C_70_(OCH_3_)_10_, C_70_(OCH_3_)_10_[C(COOEt)_2_], and C_70_(OCH_3_)_10_[C(COOEt)_2_]_2_.

The fluorescent decay profiles of the C_70_(OCH_3_)_10_, C_70_(OCH_3_)_10_[C(COOEt)_2_], and C_70_(OCH_3_)_10_[C(COOEt)_2_]_2_ in chloroform were recorded using the time-correlated single-photon counting (TCSPC) method. The fluorescence lifetime of the C_70_(OCH_3_)_10_ was described by a single-exponential component with τ = 1.16 ns. However, the fluorescence lifetime of the C_70_(OCH_3_)_10_[C(COOEt)_2_] (τ = 1.99 ns) was described by double-exponential components with τ_1_ = 1.18 ns (70.9%) and τ_2_ = 3.95 ns (29.1%). Similarly, the fluorescence lifetime of the C_70_(OCH_3_)_10_[C(COOEt)_2_]_2_ (τ = 1.82 ns) was also described by bi-exponential components with τ_1_ = 1.18 ns (72.0%) and τ_2_ = 3.44 ns (28.0%) (Table 1). As shown in Figure 4, the fluorescence lifetimes of the C_70_(OCH_3_)_10_[C(COOEt)_2_] and C_70_(OCH_3_)_10_[C(COOEt)_2_]_2_ were slightly longer than those of the C_70_(OCH_3_)_10_, which implies that the number of adducts on fullerene can influence the fluorescence lifetime of fullerene derivatives [27]. Fullerene derivatives with more adducts may have higher fluorescence quantum yields and longer fluorescence lifetimes. Therefore, multi-functionalization is a promising strategy to improve the fluorescence of fullerene derivatives.

**Figure 5:**
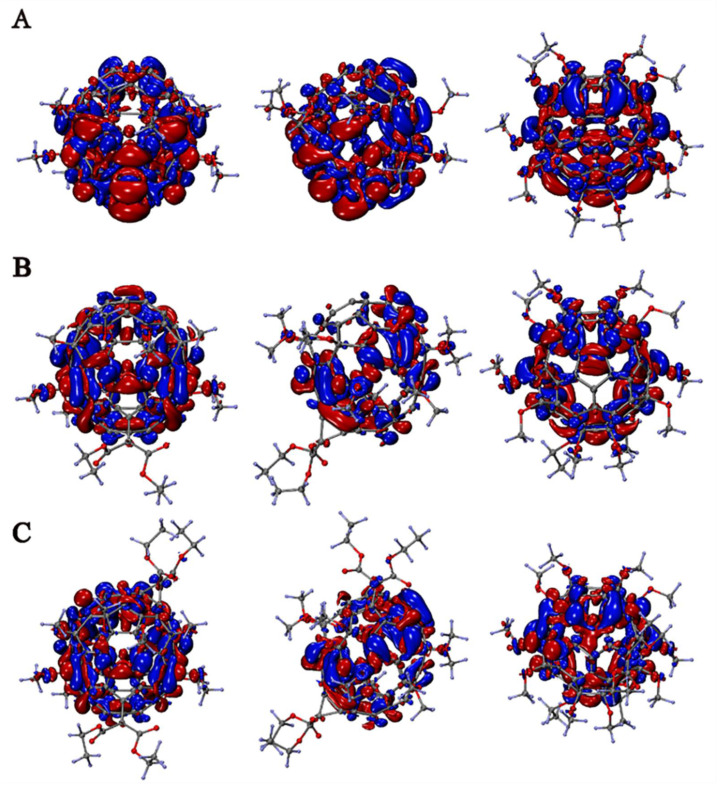
TD-DFT computed difference S_1_/S_0_ electronic densities of C_70_(OCH_3_)_10_ (**A**), C_70_(OCH_3_)_10_[C(COOEt)_2_] (**B**), and C_70_(OCH_3_)_10_[C(COOEt)_2_]_2_ (**C**). Positive and negative parts are red and blue, respectively. Each molecule is shown in three orientations: front view, side view, and top view.

To gain insight into the mechanisms of the fluorescence enhancements, we carried out theoretical calculations. Generally, the compounds with high fluorescence quantum yields had large S_1_–T_1_ energy gaps. Furthermore, the larger S_1_–T_1_ energy gaps appeared when the excitation was more localized. As shown in Figure 5, the difference S_1_/S_0_ electronic densities of the C_70_(OCH_3_)_10_, C_70_(OCH_3_)_10_[C(COOEt)_2_], and C_70_(OCH_3_)_10_[C(COOEt)_2_]_2_ were computed through TD-DFT. The excitations of the C_70_(OCH_3_)_10_[C(COOEt)_2_] and C_70_(OCH_3_)_10_[C(COOEt)_2_]_2_ were similar and spatially localized in the same fragment of the molecule, which meant a large S_1_–T_1_ energy gap. However, the large spatial extension led to a small S_1_–T_1_ energy gap, as with the C_70_(OCH_3_)_10_. Therefore, the further functionalization of the C_70_(OCH_3_)_10_ increased the S_1_–T_1_ energy gap, reducing the intersystem crossing efficiency, resulting in the higher fluorescence quantum yield of the C_70_ derivatives.

## 4. Conclusions

In summary, two multi-functionalized C_70_ derivatives, C_70_(OCH_3_)_10_[C(COOEt)_2_] and C_70_(OCH_3_)_10_[C(COOEt)_2_]_2_, were synthesized from C_70_(OCH_3_)_10_ by Bingel–Hirsch reaction with high selectivity. Compared with the C_70_(OCH_3_)_10_, the UV-vis absorption and fluorescence of both the C_70_(OCH_3_)_10_[C(COOEt)_2_] and the C_70_(OCH_3_)_10_[C(COOEt)_2_]_2_ were blue-shifted due to the decrease in the π-conjugated system of the C_70_. Moreover, the C_70_(OCH_3_)_10_[C(COOEt)_2_] and C_70_(OCH_3_)_10_[C(COOEt)_2_]_2_ showed blue fluorescence, and their fluorescence quantum yield was about ten times higher than that of the C_70_(OCH_3_)_10_. The TD-DFT calculations indicated that the multi-functionalization of the C_70_ increased the S_1_–T_1_ energy gap, reducing the intersystem crossing efficiency, resulting in the higher fluorescence quantum yield of the C_70_ derivatives. The results reveal that multi-functionalization is an effective strategy to improve the fluorescence of fullerene derivatives, providing novel organic electronic materials for organic light-emitting diodes.

## Figures and Tables

**Table 1:** Fluorescence lifetimes of C_70_(OCH_3_)_10_, C_70_(OCH_3_)_10_[C(COOEt)_2_] and C_70_(OCH_3_)_10_[C(COOEt)_2_]_2_. The values in parentheses represent the fractions of each kinetic lifetime.

	τ_1_ (ns)	τ_2_ (ns)	τ (ns)	QY (%)
C_70_(OCH_3_)_10_	1.16 (100%)		1.16	0.25
C_70_(OCH_3_)_10_[C(COOEt)_2_]	1.18 (70.9%)	3.95 (29.1%)	1.99	1.94
C_70_(OCH_3_)_10_[C(COOEt)_2_]_2_	1.18 (72.0%)	3.44 (28.0%)	1.82	2.30

## Data Availability

The data presented in this study are available in the article and Supplementary Materials.

## Supplementary Materials

The following supporting information can be downloaded at: https://www.mdpi.com/article/10.3390/nano12091426/s1. Figure S1: ^1^H NMR spectrum (500 MHz, CDCl_3_) of C_70_(OCH_3_)_10_[C(COOEt)_2_]; Figure S2: ^13^C NMR spectrum (500 MHz, CDCl_3_) of C_70_(OCH_3_)_10_[C(COOEt)_2_]; Figure S3: ESI-FT-ICR-HRMS spectra of C_70_(OCH_3_)_10_[C(COOEt)_2_], Figure S4: COSY spectra of C_70_(OCH_3_)_10_[C(COOEt)_2_]; Figure S5: HSQC of C_70_(OCH_3_)_10_[C(COOEt)_2_]; Figure S6: ^1^H NMR spectrum (500 MHz, CDCl_3_) of C_70_(OCH_3_)_10_[C(COOEt)_2_]_2_; Figure S7: ^13^C NMR spectrum (500 MHz, CDCl_3_) of C_70_(OCH_3_)_10_[C(COOEt)_2_]_2_; Figure S8: ESI-FT-ICR-HRMS spectra of C_70_(OCH_3_)_10_[C(COOEt)_2_]_2_; Figure S9: COSY spectra of C_70_(OCH_3_)_10_[C(COOEt)_2_]_2_; Figure S10: HSQC spectra of C_70_(OCH_3_)_10_[C(COOEt)_2_]_2_; Table S1: Crystallographic data for C_70_(OCH_3_)_10_[C(COOEt)_2_]; Figure S11: Natural Population Analysis (NPA) charge distribution of C_70_(OCH_3_)_10_ (A), C_70_(OCH_3_)_10_[C(COOEt)_2_]-I (B), C_70_(OCH_3_)_10_[C(COOEt)_2_]-II (C), C_70_(OCH_3_)_10_[C(COOEt)_2_]-III (D). And C_70_(OCH_3_)_10_ is shown in three orientations front view, top view and bottom view (E); Figure S12: Electrostatic potentials on the 0.001 a.u. molecular surfaces of C_70_(OCH_3_)_10_ (A), C_70_(OCH_3_)_10_[C(COOEt)_2_] (B) and C_70_(OCH_3_)_10_[C(COOEt)_2_]_2_ (C), calculated at B3LYP-D3BJ/6-31G(d, p) level with toluene solvent; Figure S13: Molecular orbitals (HOMO-1, HOMO, LUMO, and LUMO+1) of C_70_(OCH_3_)_10_ (A), C_70_(OCH_3_)_10_[C(COOEt)_2_] (B) and C_70_(OCH_3_)_10_[C(COOEt)_2_]_2_ (C) calculated at B3LYP-D3BJ/6-31G(d, p) level, in toluene; Figure S14: The most favorable structure of C_70_(OCH_3_)_10_[C(COOEt)_2_]_2_. References [31,32,33,34,35,36,37,38,39,40] are cited in supplementary materials.
