# Supplementary material for: Synthesis and Fluorescent Properties of Multi-Functionalized C70 Derivatives of C70(OCH3)10[C(COOEt)2] and C70(OCH3)10[C(COOEt)2]2
Source: Nanomaterials (Basel). 2022 Apr 22;12(9):1426. doi: 10.3390/nano12091426 (PMC9099540; doi:10.3390/nano12091426)
Supplement: Supplementary file 1 [file nanomaterials-12-01426-s001.zip › nanomaterials-1679915-supplementary.pdf]

Supplementary Materials

Synthesis and Fluorescent Properties of Multi-Functionalized C_{70} Derivatives of $C_{70}(OCH_3)_{10}[C(COOEt)_2]$ and $C_{70}(OCH_3)_{10}[C(COOEt)_2]_2$

Ke Luan [1], Lu Wang [1], Fang-Fang Xie [1], Bin-Wen Chen [1], Zuo-Chang Chen [1], Lin-Long Deng [2,*], Su-Yuan Xie [1] and Lan-Sun Zheng [1]

[1] State Key Laboratory for Physical Chemistry of Solid Surfaces, Collaborative Innovation Center of Chemistry for Energy Materials, Department of Chemistry, College of Chemistry and Chemical Engineering, Xiamen University, Xiamen 361005, China; luanke9411@163.com (K.L.); 20520201151919@stu.xmu.edu.cn (L.W.); fangfangxie0707@163.com (F.-F.X.); 2014236899@qq.com (B.-W.C.); zcchem@126.com (Z.-C.C.); syxie@xmu.edu.cn (S.-Y.X.); lszheng@xmu.edu.cn (L.-S.Z.)

[2] Pen-Tung Sah Institute of Micro-Nano Science and Technology, Xiamen University, Xiamen 361005, China

* Correspondence: denglinlong@xmu.edu.cn

Characterization of C_{70} derivatives:

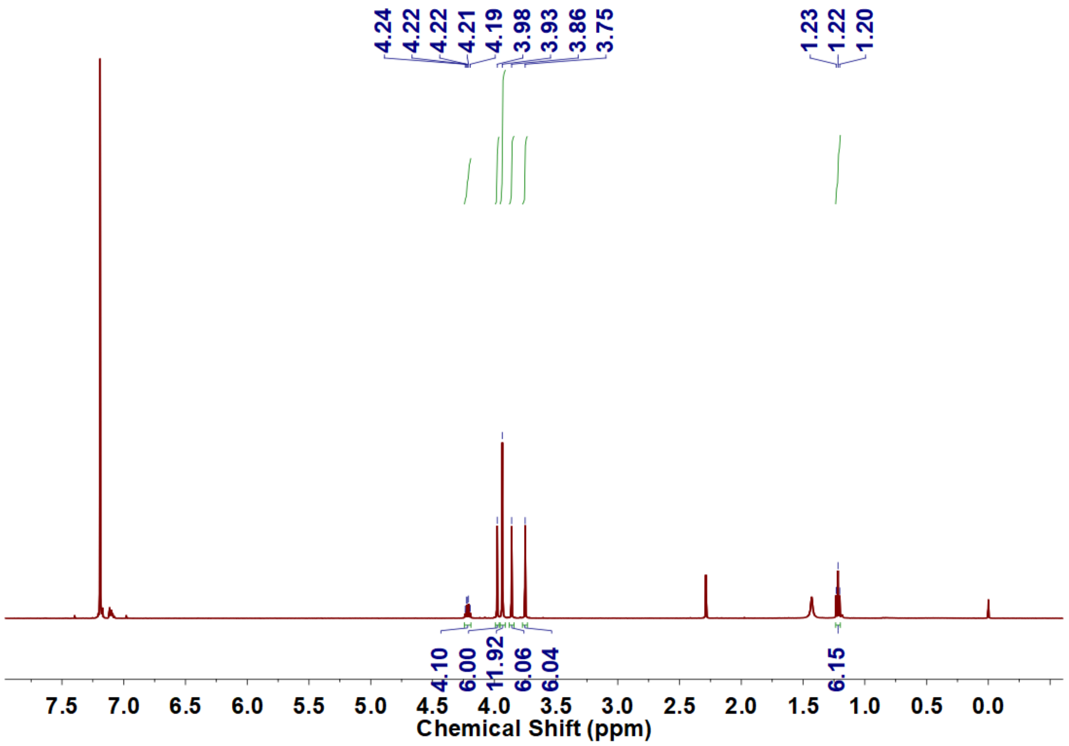

Figure S1. ^{1}H NMR spectrum (500 MHz, $CDCl_3$) of $C_{70}(OCH_3)_{10}[C(COOEt)_2]$.

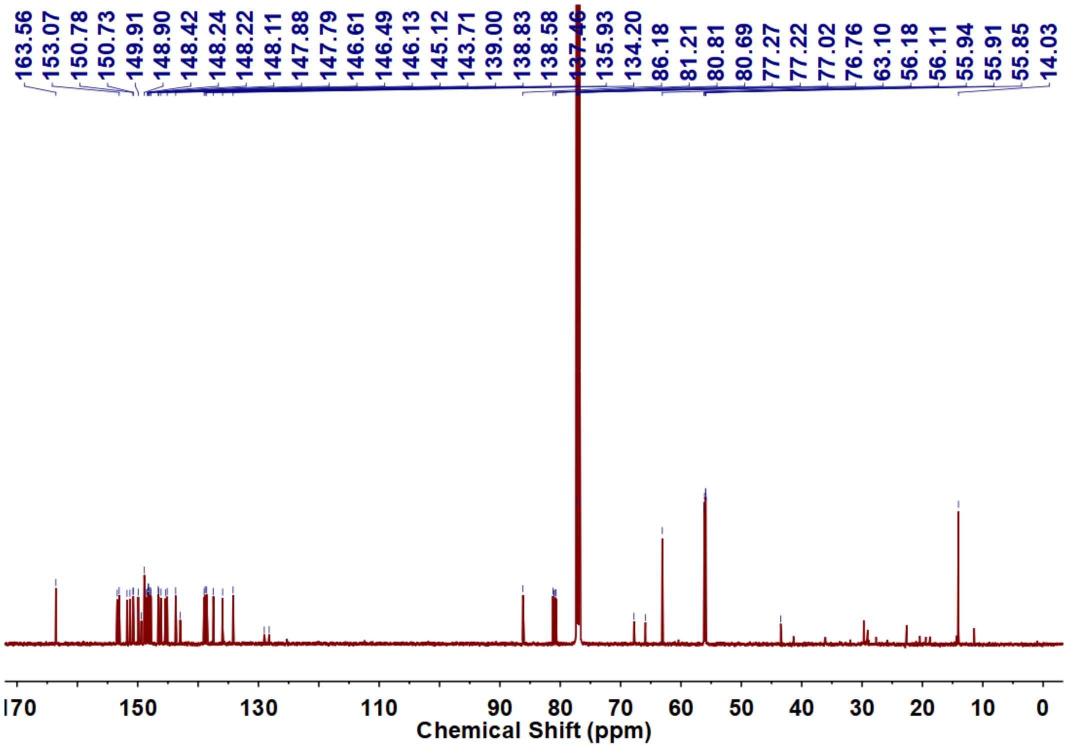

Figure S2. ^{13}C NMR spectrum (500 MHz, $CDCl_3$) of $C_{70}(OCH_3)_{10}[C(COOEt)_2]$.

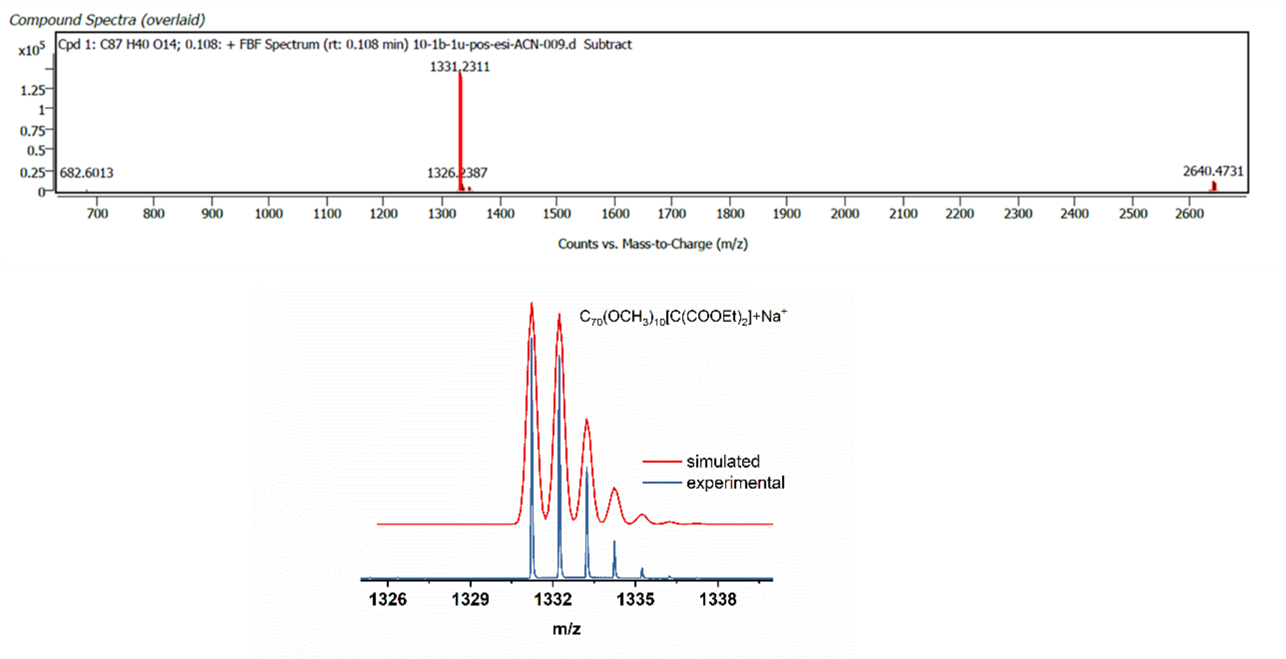

Figure S3. ESI-FT-ICR-HRMS spectra of $C_{70}(OCH_3)_{10}[C(COOEt)_2]$. The calculated isotopic distribution pattern of peak is represented in red. The mass spectra of $C_{70}(OCH_3)_{10}[C(COOEt)_2]$ is represented in blue.

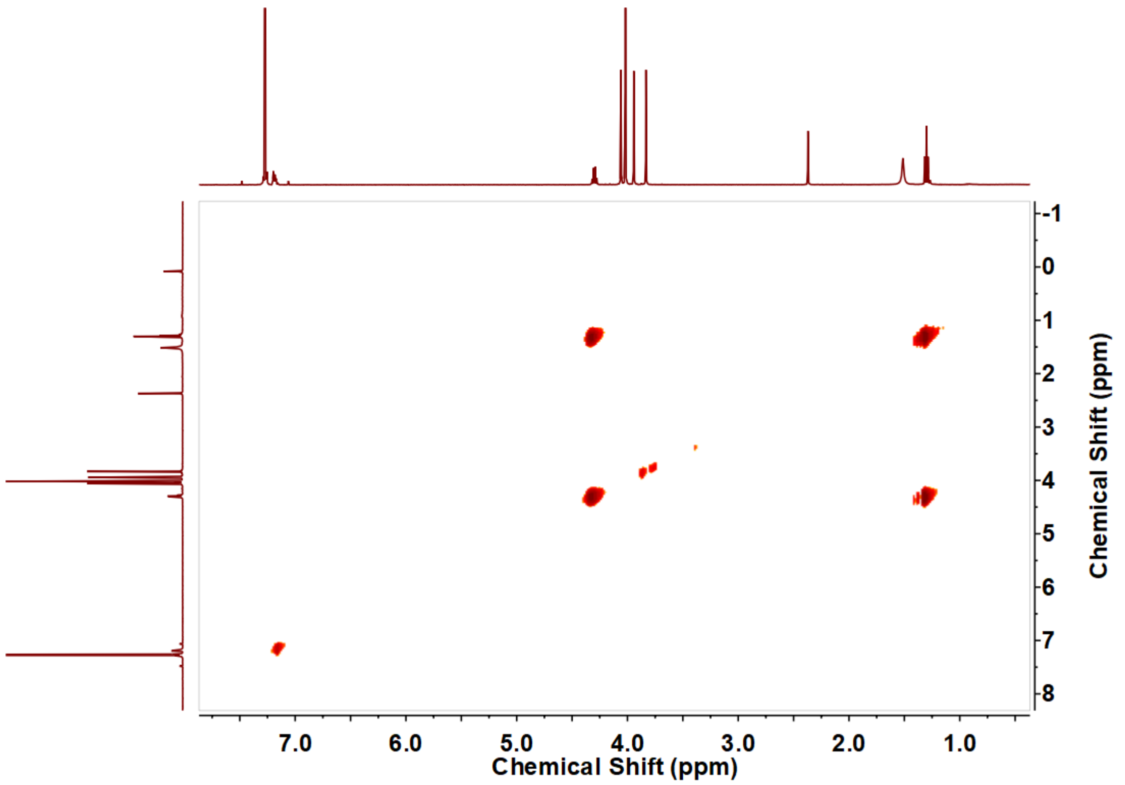

Figure S4. COSY spectra of $C_{70}(OCH_3)_{10}[C(COOEt)_2]$.

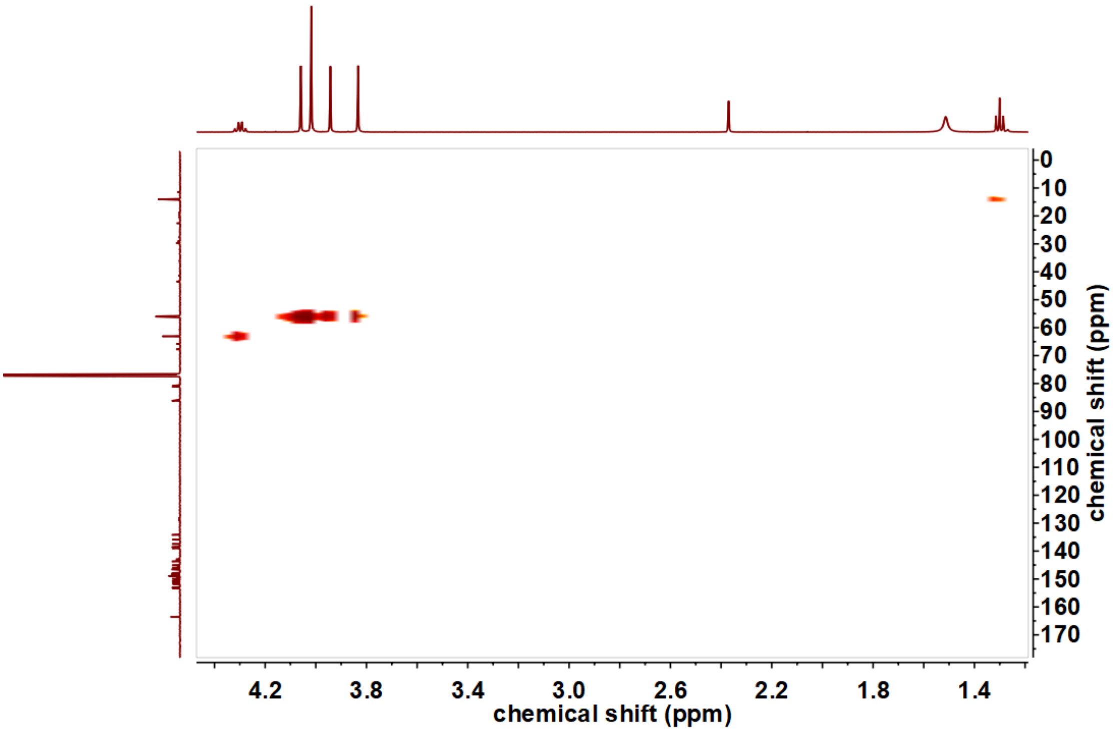

Figure S5. HSQC spectra of $C_{70}(OCH_3)_{10}[C(COOEt)_2]$.

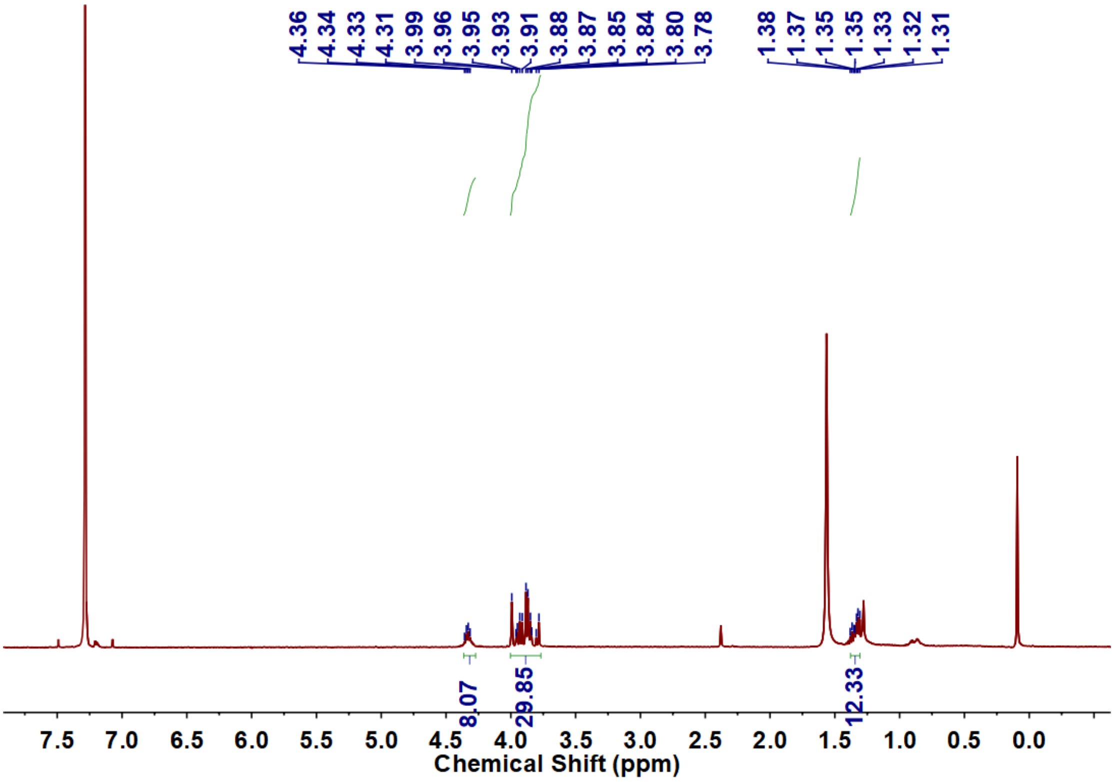

Figure S6. ^{1}H NMR spectrum (500 MHz, $CDCl_3$) of $C_{70}(OCH_3)_{10}[C(COOEt)_2]_2$.

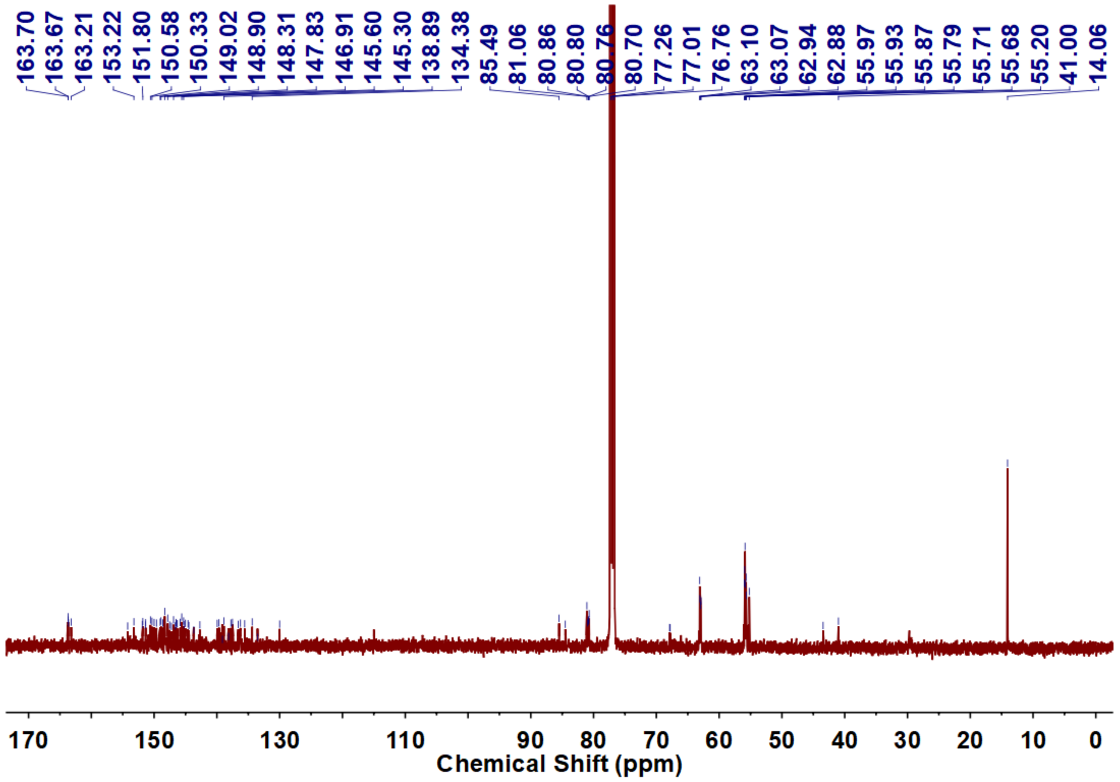

Figure S7. ^{13}C NMR spectrum (500 MHz, $CDCl_3$) of $C_{70}(OCH_3)_{10}[C(COOEt)_2]_2$.

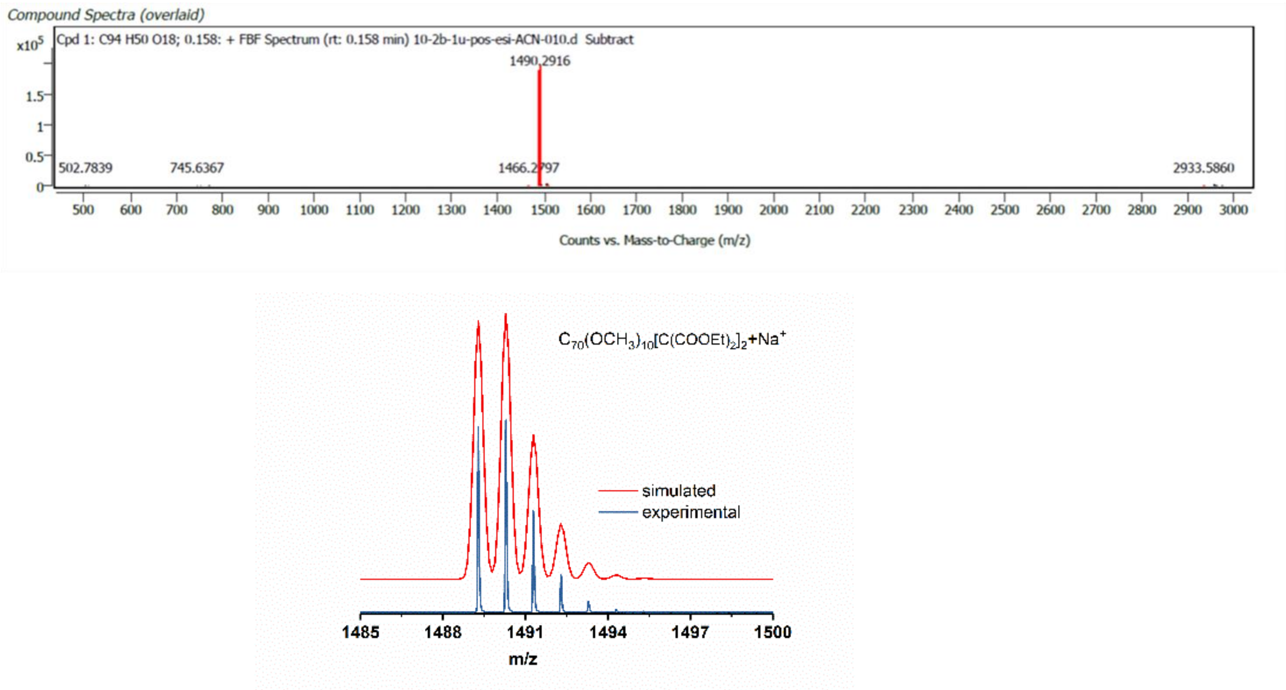

Figure S8. ESI-FT-ICR-HRMS spectra of $C_{70}(OCH_3)_{10}[C(COOEt)_2]_2$. The calculated isotopic distribution pattern of peak is represented in red. The mass spectra of $C_{70}(OCH_3)_{10}[C(COOEt)_2]_2$ is represented in blue.

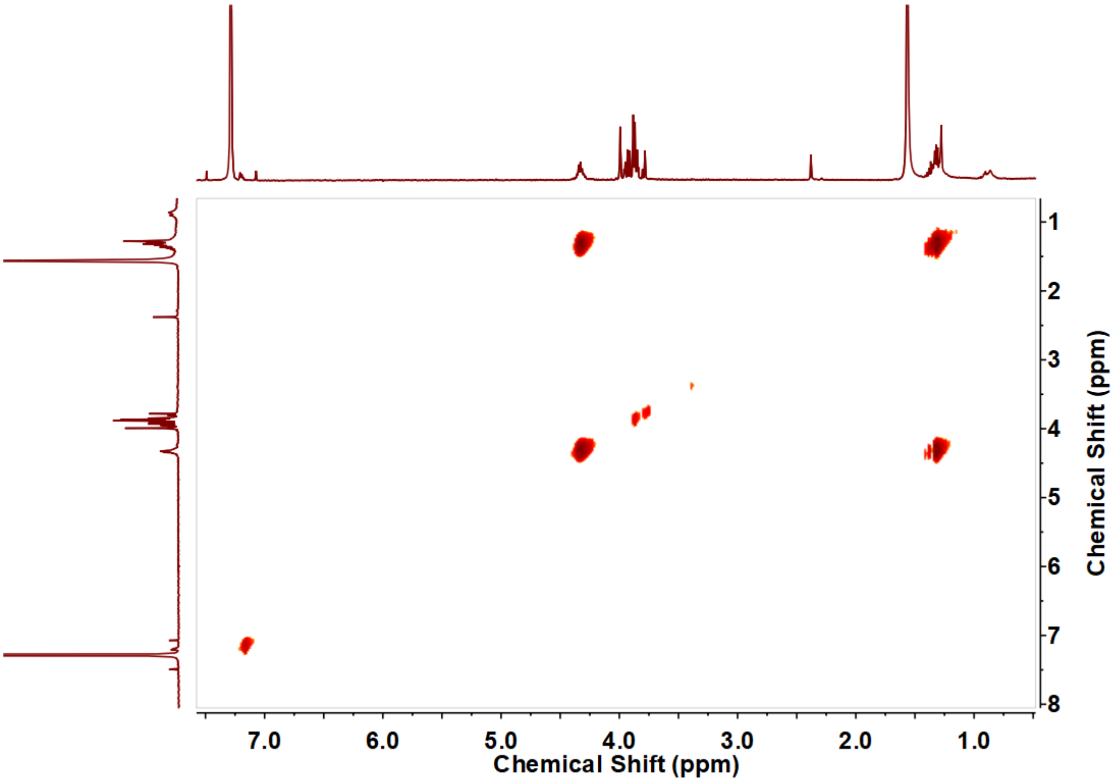

Figure S9. COSY spectra of $C_{70}(OCH_3)_{10}[C(COOEt)_2]_2$.

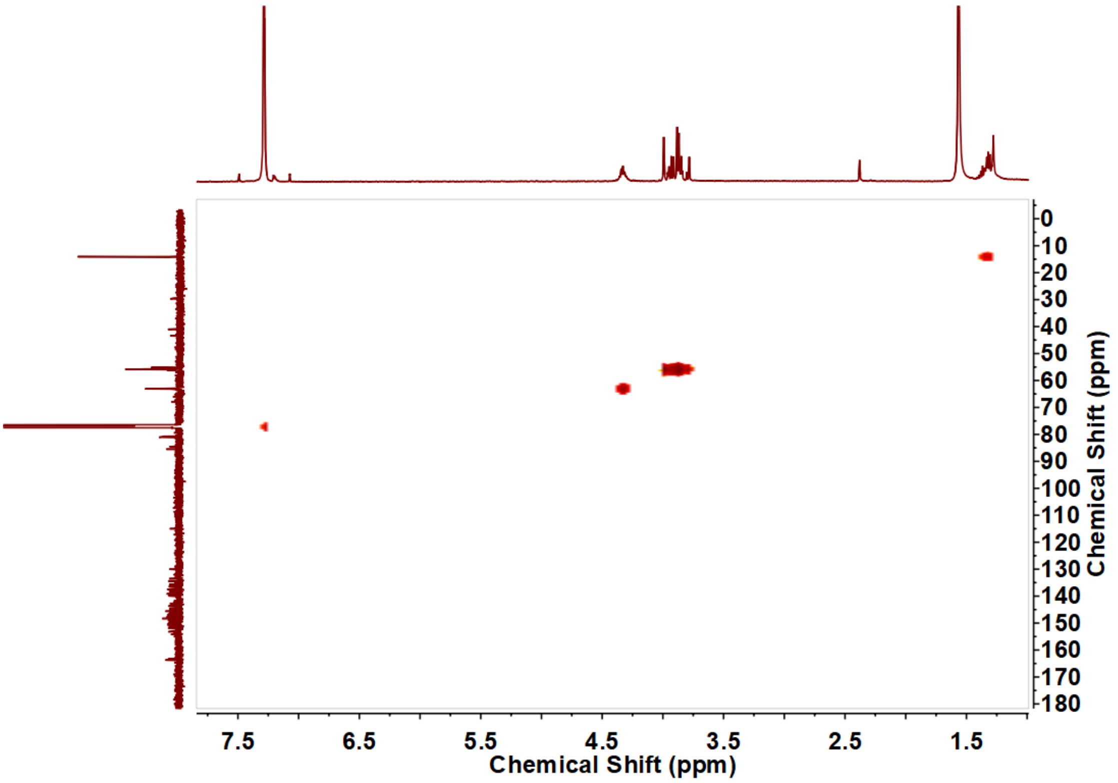

Figure S10. HSQC spectra of $C_{70}(OCH_3)_{10}[C(COOEt)_2]_2$.

Crystallographic information for $C_{70}(OCH_3)_{10}[COOEt_2]$.

X-ray diffraction analysis of $C_{70}(OCH_3)_{10}[COOEt_2]•C_7H_8$**.** The yellow block crystals of $C_{70}(OCH_3)_{10}[COOEt_2]•C_7H_8$ suitable for X-ray analysis were obtained by slowly diffusing cyclohexane into a toluene solution of $C_{70}(OCH_3)_{10}[COOEt_2]$ at low temperatures. X-ray diffraction data were collected on an XtaLAB Synergy R diffractometer with a Cu Kα (λ = 1.54184 Å) X-ray source at 100 K. The structure was solved by an intrinsic phasing method and refined using full-matrix least-squares based on F^2 with the programs SHELXT and SHELXL-2015 [31] within OLEX2 [30], respectively. CCDC 2162768 contains the supplementary crystallographic data of $C_{70}(OCH_3)_{10}[COOEt_2]•C_7H_8$. This data can be obtained free of charge from the Cambridge Crystallographic Data Centre via www.ccdc.cam.ac.uk/data_request/cif. The refined CIF was checked at the website 'http://checkcif.iucr.org/'. A summary of the crystallographic data is shown in Table S1.

Table S1. Crystallographic data for $C_{70}(OCH_3)_{10}[C(COOEt)_2]$

Formula	$C_{87}H_{40}O_{14}$
Crystal system	triclinic
Space group	*P*-1
a, Å	13.7077(3)
b, Å	13.7352(3)
c, Å	17.6171(4)
α, deg	82.321(2)
β, deg	68.644(2)
γ, deg	74.540(2)
V, Å^3	2974.91(12)
Z	2
T, K	100
Crystal description	block
Crystal size, mm^3	0.4×0.3×0.3

2θmin, 2θmax, deg	5.39, 134.256
no. refl. Measured ($I > 2δ(I)$)	10297
no. parameters	996
R_1, wR_2 (all data)	0.0487, 0.1087
R_1, wR_2 ($I > 2δ(I)$)	0.0415, 0.1047
GOF on F^2	1.058

Theoretical calculations:

All DFT and TD-DFT theoretical calculations were performed with Gaussian16 software package, at the B3LYP-D3BJ/6-31G(d,p) level [32–36]. Toluene Solvent effects were considered by the polarizable continuummodel (PCM) [36]. Isosurface are visually demonstrated using VMD software [37].

The nucleophilic cycloaddition Bingel-Hirsch reaction has significant regioselectivity. Generally, the preferred addition sites are on [35] bond and with high pyramidalization angles [38,39]. In order to explore the possible addition sites of diethyl bromomalonate group on $C_{70}(OCH_3)_{10}$, we performed DFT calculations at the B3LYP-D3BJ/6-31G(d,p) level. As the electrostatic potential shown in Figure S12, 1'~10' atoms (bottom view) has more positive surface potential than 1~10 atoms (top view), which means that the 1'-10' atoms is easier to be attacked by the diethyl bromomalonate anion (Figure S11E). According to the natural population analysis (NPA), four atoms (indices 6', 10', 6 and 9, marked in red) are positively charged (Figure S11A). Further molecular orbitals analyses indicate that the LUMO of $C_{70}(OCH_3)_{10}$ is mainly located on atoms 1',2',5', 6', 9' and 10', and atoms 1' and 10' contributes the largest to this unoccupied orbital over the bottom atoms 1'-10' (Figure S13). It means that 1' and 10' atoms (bond **I**) are more likely to accept electrons from the diethyl bromomalonate anion. Moreover, compare with other three positively charged atoms, atoms 10' has the lowest steric hindrance. Therefore, atom 10' shows

the best reaction activity. For $C_{70}(OCH_3)_{10}[C(COOEt)_2]$, around $-C(COOEt)_2$ group atoms with higher steric hindrance show the most positively charged state, and the second most positively charged atoms are on the other side (Figure S11). In terms of electronic and steric factors, the first addition (bond **I**) at bottom view atoms results that the top view atoms become the second most favorable site (bond **III**). Conversely, the first addition at top view atoms (bond **III**) results in the bottom view atoms becoming the second most favorable site (bond **I**). Similar C_{70} derivatives were prepared by Ovchinnikova et al. [40,28] show the same regioselectivity.

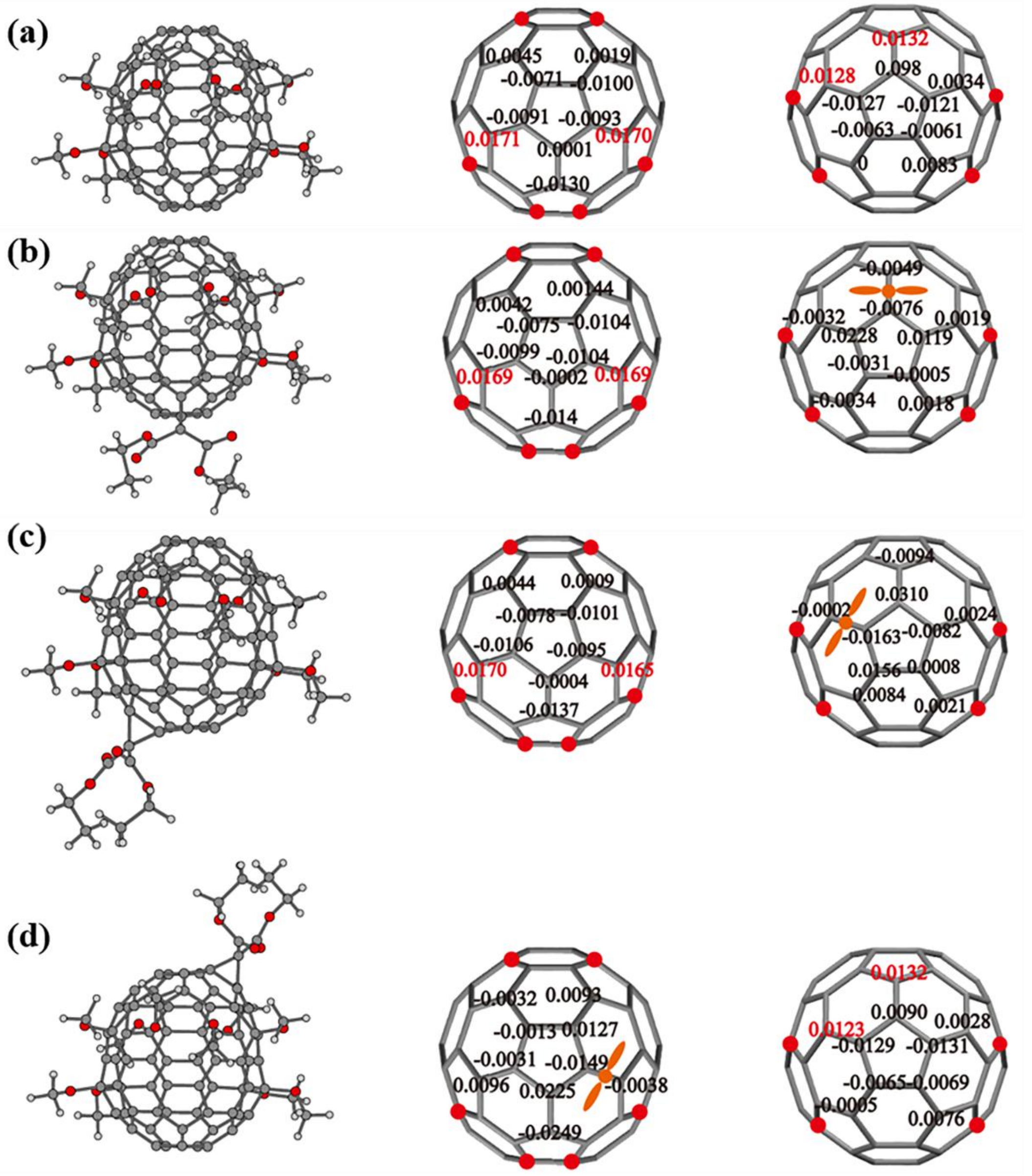

(e)

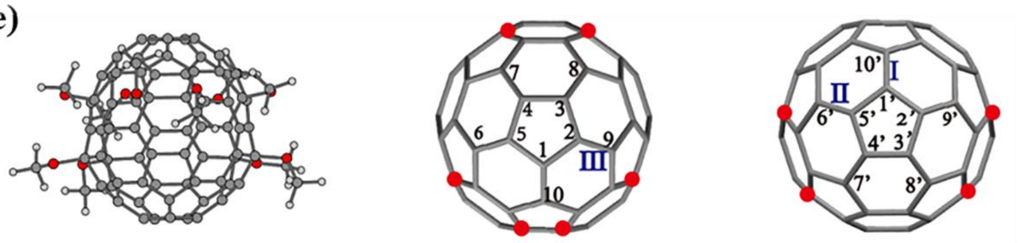

Figure S11. Natural Population Analysis (NPA) charge distribution of $C_{70}(OCH_3)_{10}$ (a), $C_{70}(OCH_3)_{10}[C(COOEt)_2]$-I (b), $C_{70}(OCH_3)_{10}[C(COOEt)_2]$-II (c), $C_{70}(OCH_3)_{10}[C(COOEt)_2]$-III (d). And $C_{70}(OCH_3)_{10}$ is shown in three orientations front view, top view and bottom view (e). In the top view and bottom view, indices 1-10,1'-10' denote atoms at which nucleophilic regent may attack. Indices I-III denote bonds at which cycloaddition may occur, red circles denote -OCH_3 groups, orange pattern denote attached diethyl bromomalonate group. In the top view and bottom view, indices 1-10,1'-10' denote atoms at which nucleophilic regent may attack, indices I-III denote bonds at which cycloaddition may occur, red circles denote -OCH_3 groups, orange pattern denote attached diethyl bromomalonate group.

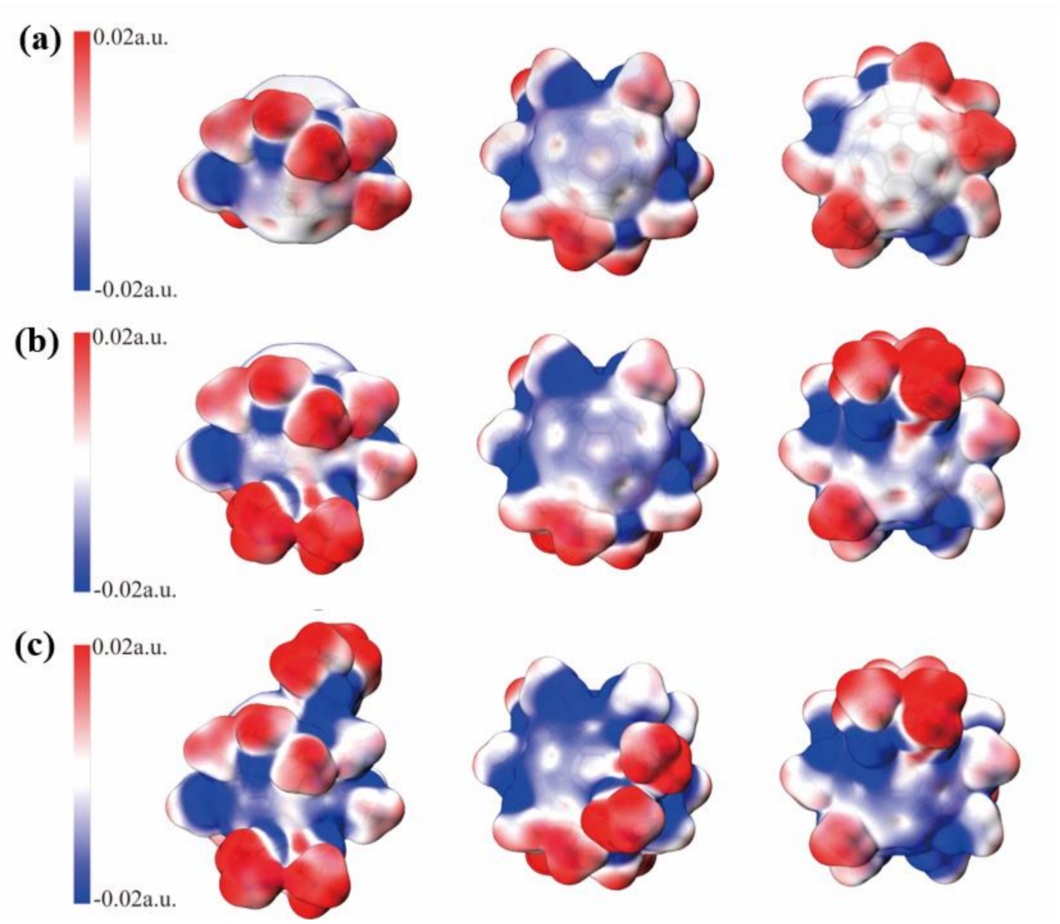

Figure S12. Electrostatic potentials on the 0.001 a.u. molecular surfaces of $C_{70}(OCH_3)_{10}$ (a), $C_{70}(OCH_3)_{10}[C(COOEt)_2]$ (b) and $C_{70}(OCH_3)_{10}[C(COOEt)_2]_2$ (c), calculated at B3LYP-D3BJ/6-31G(d, p) level with toluene solvent. Each molecule is shown in three orientations front view, top view and bottom view.

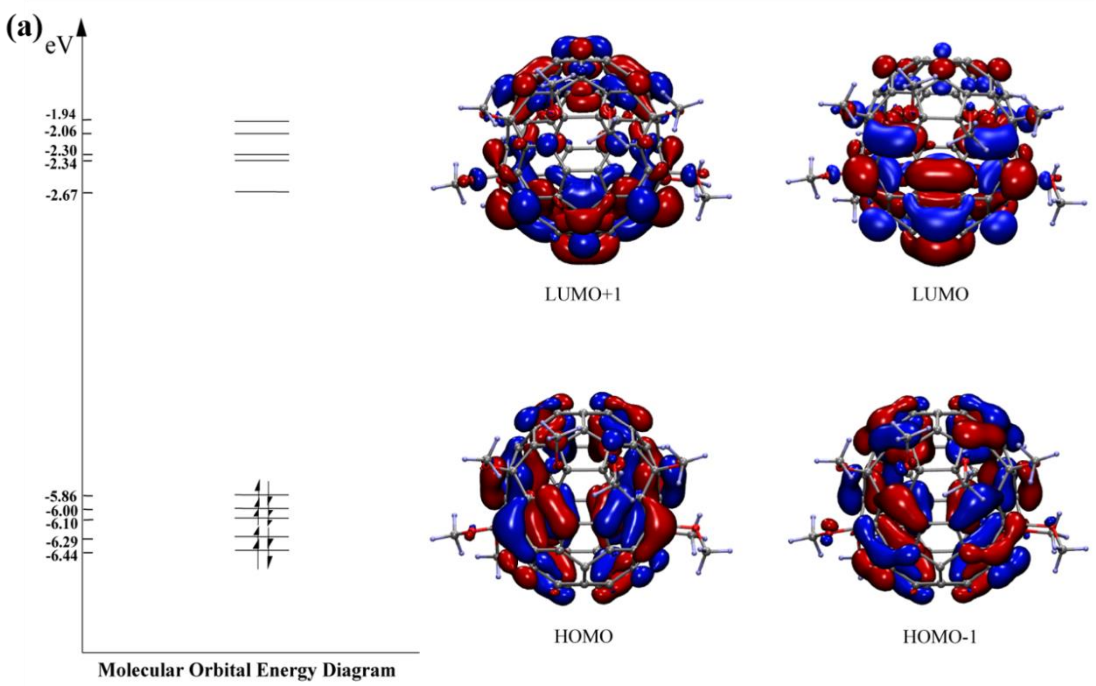
(a)
eV
-1.94
-2.06
-2.30
-2.34
-2.67
-5.86
-6.00
-6.10
-6.29
-6.44
Molecular Orbital Energy Diagram
LUMO+1
LUMO
HOMO
HOMO-1

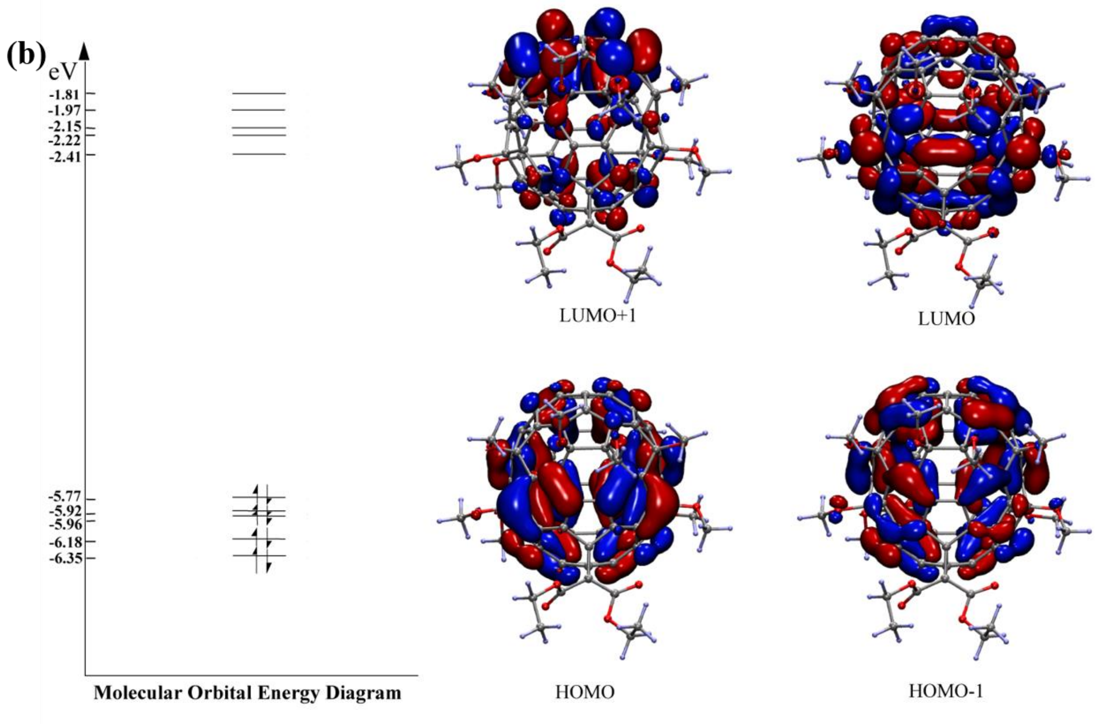
(b)
eV
-1.81
-1.97
-2.15
-2.22
-2.41
-5.77
-5.92
-5.96
-6.18
-6.35
Molecular Orbital Energy Diagram
LUMO+1
LUMO
HOMO
HOMO-1

(c)

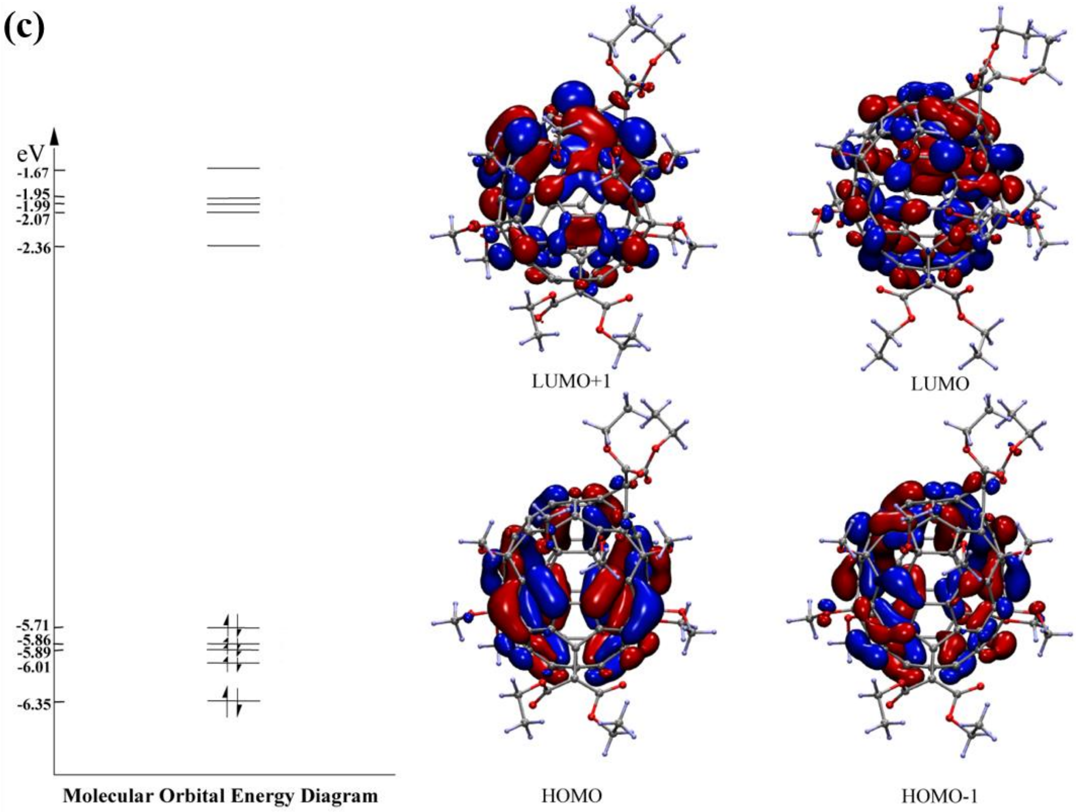

Figure S13. Molecular orbitals (HOMO-1, HOMO, LUMO, and LUMO+1) of $C_{70}(OCH_3)_{10}$ (a), $C_{70}(OCH_3)_{10}[C(COOEt)_2]$ (b) and $C_{70}(OCH_3)_{10}[C(COOEt)_2]_2$ (c) calculated at B3LYP-D3BJ/6-31G(d, p) level, in toluene.

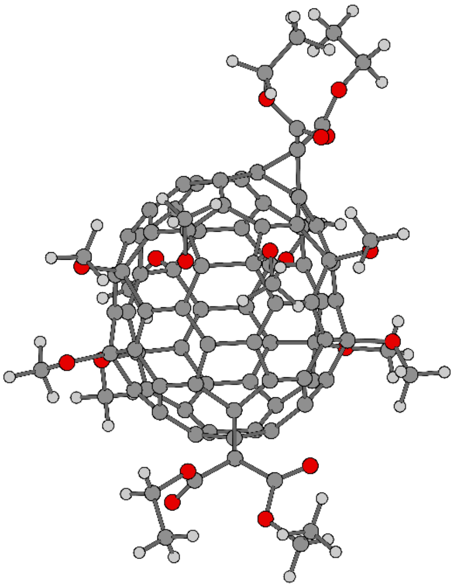

Figure S14. The most favorable structure of $C_{70}(OCH_3)_{10}[C(COOEt)_2]_2$.
